# Moxibustion therapy for treating patients with postpartum urinary retention: A protocol for systematic review and meta-analysis

**DOI:** 10.1097/MD.0000000000025683

**Published:** 2021-04-30

**Authors:** Tianjiao Li, Xin Hui, Hao Wang, Yao Lin, Baixiao Zhao

**Affiliations:** Beijing University of Chinese Medicine, Chaoyang, Beijing, China.

**Keywords:** complementary medicine, moxibustion therapy, postpartum urinary retention, protocol, systematic review

## Abstract

**Background:**

Postpartum urinary retention (PUR) is one of the most common complications after parturition which affect women's recovery after childbirth. Many clinical trials have shown that moxibustion, a traditional Chinese medicine therapy, is effective in treating PUR. But its effectiveness has not been evaluated scientifically and systematically. Therefore, this review aims to evaluate the safety and effectiveness of moxibustion therapy in treating patients with PUR.

**Methods:**

We will search the following electronic databases, regardless of publication status and languages, from their respective inception dates to February 2021: the Cochrane Central Register of Controlled Trails, Pubmed, EMBASE, China National Knowledge Infrastructure, Chinese Biomedical Literature Database, Chinese Scientific Journal Database, and Wan-Fang Database. Clinical randomized controlled trials (RCTs) related to moxibustion therapy for treating PUR will be included. Study selection, data collection, and quality assessment will be independently conducted by 2 researchers. For data synthesis, we will select either the fixed-effects or random-effects model according to heterogeneity assessment. Cure rates and postvoid residual volume (PVRV) will be the primary outcomes. The total effective rate and first urination time will be the second outcomes. Review Manager Software (RevMan) V.5.3 will be used if it is appropriate for meta-analysis. Otherwise, a systematic narrative synthesis will be conducted. The results will be presented as risk ratio (RR) with 95% confidence interval (CI) for dichotomous data and weight mean difference (WMD) or standard mean difference (SMD) 95% CI for continuous data.

**Trial registration number:**

INPLASY 202140037.

## 1 Introduction

Postpartum urinary retention (PUR) is one of the most common complications after parturition. It is defined that women cannot urinate spontaneously within 6 hours after vaginal delivery or the removal of an indwelling catheter in the case of cesarean section. Women undergoing incomplete urination after childbirth (which refers to postvoid residual volume more than 150 ml after the first spontaneous urination) are also diagnosed as covert PUR.^[1]^ Main independent risk factors associated with PUR^[2]^ are episiotomy, epidural analgesia, primiparity, instrumental delivery, vulvar edema or perineal hematoma,^[3]^ and the duration of the second stage of labor. According to researches, the incidence of PUR is ranging from 1.7% to 17.9%.^[4]^ PUR may lead to abdominal pain, and increase vaginal hemorrhage as well as urinary tract infection, which, as a consequence, may result in the raised risk of postpartum depression, extended length of hospitalization and mounting costs.

There are no clinical guidelines on the treatment for PUR. The routine management of postpartum voiding and PUR commonly includes induction, medication and catheterization.^[5]^ Nevertheless, the effects of induction, such as simulating the sound of running water, massaging over the bladder region, taking warm baths, and rinsing perineum, are individually different, that is, not ideal to every patient.^[6]^ Medications and catheterization will be applied if such non-pharmaceutical treatments are ineffective. These are standard treatments but urethral catheterization can increase the risk of urinary tract infection.^[7]^

Moxibustion, a traditional therapy in Chinese medicine, is a viable supplemental replacement therapy in China, Japan, Korea, and many other countries and regions. Years of application experience and clinical trials have shown that moxibustion can promote the recovery of postpartum urinary retention.^[8–14]^ Moxibustion therapy generally applied for treating PUR in clinical practice involve either traditional moxibustion with pure moxa floss (moxa stick or cone), mixed moxa floss with Chinese herbs (thunder-fire moxibustion),^[10]^ or combined moxibustion with acupuncture or electric acupuncture.^[15,16]^ According to Traditional Chinese Medicine theory, moxibustion therapy can warm the channels and promote qi circulation. Studies have shown that the heat of moxibustion can relieve the spasm of urethral sphincter, promote the recovery of bladder sensory function, excite the peripheral nerve and bladder sphincter, promote bladder contraction, thereby promoting excretion of urine.^[17]^

Moxibustion is a non-invasive therapy, which is easy to apply. It is low-cost, painless and at the same time has less adverse reactions compared to medications and catheterization. As there is no critically designed systematic review to assess the effectiveness and safety of moxibustion for PUR, we aim to evaluate whether moxibustion is a good choice for PUR patients, and whether it is so effective that it can be a substitution therapy compared to other therapies.

## 2 Methods

### 2.1 Study registration

This systematic review protocol was registered on INPLASY (registration number is INPLASY 202140037) and is available in full on the inplasy.com (https://doi.org/10.37766/inplasy2021.4.0037). This protocol has been drafted under the guidance of the Preferred Reporting Items for Systematic Reviews and Meta-analysis Protocols (PRISMA-P) statement guidelines^[18]^ and Cochrane handbook for systematic reviews of interventions.^[19]^ This systematic review will be conducted from March 1, 2021 to March 1, 2022, when we will have all reviewers to receive a consistency training to get a basic understanding of the background, purpose, and process of the review.

### 2.2 Criteria for including studies

#### 2.2.1 Types of studies

We will include randomized controlled trials (RCTs) and randomized cross-over trials related with moxibustion in the treatment for PUR without restrictions on publication status. Quasi-randomized trials will be excluded.

#### 2.2.2 Types of participants

Patients diagnosed with PUR after parturition will be included. There will be no restrictions on age, race, educational or economic status among patients. Patients diagnosed with postoperative urinary retention, such as abdominal or pelvic surgery, will be excluded.

#### 2.2.3 Types of interventions and comparisons

Moxibustion therapy and related therapies are included as interventions in this review, such as moxa stick moxibustion, moxibustion with moxa cone, and moxibustion with moxibustioner (a device for moxibustion), heat-sensitive moxibustion, natural moxibustion, and thunder-fire moxibustion. Because we aim to evaluate the pure effect of moxibustion in the treatment for PUR, trials that involve a combination of moxibustion with other treatments like acupuncture, massage, cupping, and drugs or Chinese herbs will be excluded. The following treatment comparisons will be investigated:

1. Moxibustion compared with no treatment;
2. Moxibustion compared with placebo or sham moxibustion;
3. Moxibustion compared with other therapies.

#### 2.2.4 Types of outcome measures

##### 2.2.4.1 Primary outcomes measures

The primary outcomes will be cure rates and postvoid residual bladder volume. Postvoid residual volume under ultrasonic testing is a widely accepted measure to evaluate the bladder function.

##### 2.2.4.2 Secondary outcomes measures

The secondary outcomes will be total effective rates and first urination time. In addition, adverse events in the treatment will also be accessed in the review to evaluate the safety of moxibustion therapy.

### 2.3 Search strategy

The following electronic databases will be searched, regardless of publication status and languages, from their respective inception dates to February 2021: the Cochrane Central Register of Controlled Trails, PubMed, EMBASE, the Web of Science, China National Knowledge Infrastructure, Chinese Biomedical Literature Database, Chinese Scientific Journal Database, and Wan-Fang Database. Furthermore, we will search the conference abstracts and trial registered platforms, including Clinical Trials.gov and the Chinese Clinical Trial Registry to obtain ongoing or unpublished trials. The reference lists and the citation lists of studies meeting the inclusion criteria and relevant systematic reviews will also be searched to identify further studies for inclusion.

Two researchers (HW and YL) will search the above databases independently, and will retrieve these databases again before publication to ensure that all documents are included. A search strategy has been established according to the Cochrane handbook guidelines (Table 1). The search strategy will be applied to all the databases (equivalent search terms will be used in the Chinese databases).

**Table 1:** Search strategy used in PubMed.

Number	Search terms
1	Randomized controlled trial.pt
2	Controlled clinical trial.pt
3	Randomized. ti, ab
4	Randomly. ti, ab
5	Placebo. ti, ab
6	Trial. ti, ab
7	Groups. ti, ab
8	1 or 2–7
9	Postpartum period. Mesh
10	Postpartum. ti, ab
11	Postpartum. ti, ab
12	Postnatal. ti, ab
13	Post-nata. ti, ab
14	Puerperium. ti, ab
15	9 or 10–14
16	Postpartum period/urine. Mesh
17	Urinary retention. Mesh
18	Urinary retention. ti, ab
19	17 or 18
20	Moxibustion. Mesh
21	Moxibustion. ti, ab
22	Moxa. ti, ab
23	Mox^∗^.ti, ab
24	Mugwort. ti, ab
25	Moxibustion theropy. ti, ab
26	20 or 21–25
27	8 and 15 and 19 and 26

### 2.4 Data collection and analysis

#### 2.4.1 Selection of studies

**Figure 1:**
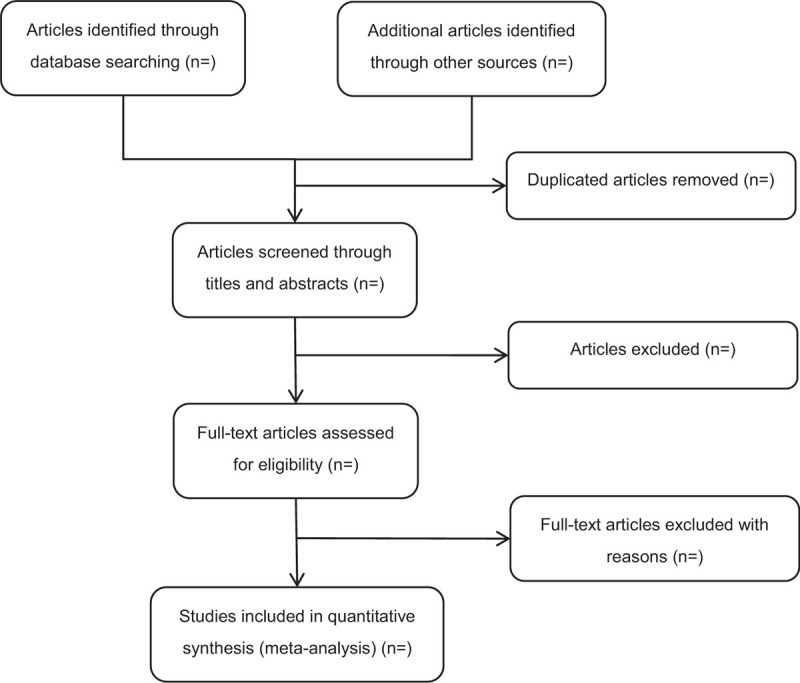
Flow diagram of study selection.

The search results selected from the databases will be entered into EndNote software (V.X9) to manage the trails and remove duplicates. Two reviewers (HW and YL) will conduct primary screening by checking the titles and abstracts of all retrieved researches to include eligible trials independently. The full text will be read, if the 2 reviewers can not identify whether the study should be included by the above content. All the articles will be divided into “included” or “excluded,” and the reason of exclusion will be recorded. Differences of opinions between the 2 review authors will be arbitrated by the third author (BXZ). The original authors of the selected articles will be contacted for clarification when necessary. The process of the selection is shown in Figure 1.

#### 2.4.2 Data extraction and management

Before data extraction, 2 review authors (TL and XH) will reach a consensus on the content of data extraction form, and then extract the data and assess the qualities of the studies independently. The following factors will be collected: general information, methods, participants, interventions, outcomes, results, adverse events and other information. A third reviewer (BXZ) will join the discussion and judge the disagreements during the course. Review Manager Software (RevMan) V.5.3 will be used for data analysis and synthesis.

#### 2.4.3 Assessment of risk of bias in included studies

The Cochrane Collaboration tool for assessing the risk of bias will be used to evaluate the methodological quality in included studies. The risk of bias of each study will be assessed by 2 authors (TL and XH) independently and disagreements will be judged by the third author (BXZ). The following aspects for risk of bias will be assessed: selection bias (random sequence generation and allocation concealment), performance bias (blinding of the participants), detection bias (blinding of the outcome assessment), attrition bias (incomplete outcome data), reporting bias (selective outcome reporting), and other sources of biases. It will be classified into 3 levels: low risk, high risk, and unclear risk.

#### 2.4.4 Measures of treatment effect

Weight mean difference (WMD) or standard mean difference (SMD) will be applied to measure the continuous data. Risk ratio (RR) will be applied to measure the dichotomous data. The estimated value and 95% confidence interval (CI) of each effect size will be given.

#### 2.4.5 Unit of analysis issues

Data that are from parallel-group studies will be extracted for analysis. As for trials with multiple observation nodes, only the end of the treatment data or the end of the follow-up data will be extracted for assessment. The individual participant in every study will be regarded as the analytical unit, and data for each outcome from each participant will be collected.

#### 2.4.6 Management of missing data

We will contact the original authors of the included studies to get the missing or incomplete data. If the data cannot be provided, we will use the available data to accomplish our analysis. Reasons of missing data will be explained and recorded

#### 2.4.7 Assessment of heterogeneity

Higgins *I*^2^ statistic test will be used to quantify heterogeneity.^[20]^ If the *I*^2^ test value is less than 50%, substantial heterogeneity will not be considered to exist in the studies. If the *I*^2^ values more than 50%, we will explore and report the possible reasons.

#### 2.4.8 Assessment of reporting biases

Funnel plots will be used to assess the reporting biases and small-study effects if the number of included studies in the meta-analysis is more than 10.^[21]^ All the eligible studies will be included for funnel plots, regardless of their methodological quality.

#### 2.4.9 Data synthesis

RevMan V.5.3 statistical software will be applied for data synthesis when meta-analysis is possible. The results of dichotomous data will be expressed as RR with 95% CI. The results of continuous data will be expressed as WMD or SMD with 95% CI. If the *I*^2^ test values less than 50%, the fixed-effects model will be used. If the *I*^2^ value is between 50% and 75%, the random effect model will be conducted for data synthesis. If the *I*^2^ value is higher than 75%, descriptive analysis will be considered. If quantitative synthesis is not appropriate such as insufficient RCTs or unidentified significant heterogeneity, we will conduct subgroup analysis or provide a systematic narrative synthesis to describe the characteristics and findings of the included trials.

#### 2.4.10 Subgroup analysis

Subgroup analysis will be conducted if significant heterogeneity exists. If the studies are adequate, subgroups of different moxibustion forms will be considered, such as thunder-fire moxibustion, herb-partitioned moxibustion, or natural moxibustion. Different forms of moxibustion will be classified as 1 group in the meta-analysis.

#### 2.4.11 Sensitivity analysis

Sensitivity analysis will be conducted to evaluate the robustness of the primary results. The principal decision nodes will include methodological quality, sample size, and the effect of missing data. The meta-analysis will be repeated and studies of lower quality will be excluded.

#### 2.4.12 Grading the quality of evidence

We will use the Grading of Recommendations Assessment, Development and Evaluation (GRADE) to evaluate the quality of evidence for all outcomes. The GRADE assessment classifies the quality of evidence into 4 levels: high, moderate, low, and very low. The following domains will be assessed: risk of bias, consistency of results, directness, precision and publication bias.

## 3 Discussion

Moxibustion therapy has been playing an important role in the history of Chinese medicine. It has been proved by clinical practice and many RCT trails to be effective on treating PUR. Currently, no systematic review related to moxibustion for PUR has been published.

Hence, this review will provide a summary of the current evidence on the effectiveness and safety of moxibustion therapy for PUR. The evaluation process will be divided into 4 sections: identification, study inclusion, data extraction, and data synthesis. We hope this review will be helpful for practitioners, patients, and health policy-makers on making decisions when dealing with PUR.

## Author contributions

**Conceptualization:** Tianjiao Li, Xin Hui.

**Data curation:** Xin Hui, Hao Wang.

**Formal analysis:** Tianjiao Li, Yao Lin.

**Methodology:** Tianjiao Li, Xin Hui, Hao Wang, Baixiao Zhao.

**Project administration:** Tianjiao Li, Xin Hui, Baixiao Zhao.

**Supervision:** Baixiao Zhao.

**Validation:** Hao Wang, Yao Lin, Xin Hui.

**Writing – original draft:** Tianjiao Li, Hao Wang.

**Writing – review & editing:** Tianjiao Li, Xin Hui, Hao Wang, Yao Lin, Baixiao Zhao.
